# Effects of phase-specific GnRH administration on ovarian functional markers, ovulation timing, and fertility in estrous-synchronized ewes

**DOI:** 10.3389/fvets.2025.1683330

**Published:** 2025-10-31

**Authors:** Jose Francisco Cox, Felipe Navarrete, Antonio Bocic, Fernando Saravia, Jesús Dorado

**Affiliations:** ^1^Faculty of Veterinary Sciences, Universidad de Concepción, Chillán, Chile; ^2^Department of Animal Medicine and Surgery, Faculty of Veterinary Medicine, Universidad de Córdoba, Córdoba, Spain

**Keywords:** estrous synchronization, GnRH, ovulation control, follicular dynamics, sheep

## Abstract

Suboptimal fecundity rates remain a major limitation of estrous synchronization (ES) protocols in sheep. This study tested the hypothesis that GnRH administration, either to promote follicular diameter homogeneity or to control ovulation timing, could improve ovarian functional outcomes to increase fecundity rates in treated ewes. Experiment 1 assessed whether GnRH administration 36 h after CIDR removal could control the timing of ovulation in ewes treated with a short-term CIDR + PGF₂*α* protocol, with or without eCG. Ewes were assigned to: CIDR + eCG (Group 1, *n* = 23), CIDR + eCG + GnRH (Group 2, *n* = 26), or CIDR + GnRH (Group 3, *n* = 24). Experiment 2 evaluated the fertility impact of the same protocols across two commercial farms (*n* = 370), using similar groupings (CIDR, CIDR + eCG, CIDR + eCG + GnRH). All ewes were naturally mated after CIDR removal. Morphological and endocrine markers were recorded to assess follicular growth, ovulation, and corpus luteum (CL) development, while fertility outcomes included pregnancy, lambing, and fecundity rates. Experiment 3 assessed whether GnRH administration during the early follicular phase (day 3) of a Synchrovine protocol could reduce follicular diameter heterogeneity at ovulation. Ewes (*n* = 45) received either PGF + PGF (controls, *n* = 23) or PGF + GnRH + PGF (*n* = 22) and were mated on day 7. GnRH shortened the interval to ovulation (*p* < 0.0001) and concentrated ovulatory timing (*p* = 0.0026) in Exp. 1. In Exp. 2, GnRH increased fecundity compared to CIDR + eCG (*p* = 0.007) and CIDR-only groups (*p* = 0.004). In Exp. 3, GnRH reduced heterogeneity in follicular diameters (*p* = 0.004) but did not affect ovulation or fertility (*p* > 0.10). These findings indicate that GnRH, when administered in the late follicular phase, improves ovulation synchrony and fertility, whereas its earlier use for follicular homogenization alters morphology but not reproductive outcomes.

## 1 Introduction

Estrous synchronization (ES) for programmed reproduction has the potential to simplify artificial insemination and lamb production protocols in intensive sheep systems. However, the widespread adoption of ES in sheep remains limited, primarily due to suboptimal fecundity rates observed after treatment. Conventional ES protocols—based on long (12–14 days) or short (6–8 days) progestogen treatments combined with equine chorionic gonadotropin (eCG) or prostaglandin F2α (PGF) protocols (e.g., Synchrovine; (1, 2))—have been extensively employed (3, 4). Nonetheless, these protocols have not consistently matched the lamb production achieved by non-synchronized ewes, limiting their practical impact in commercial settings (5, 6).

Fecundity in sheep is largely determined by the number of follicles recruited into the ovulatory wave that complete terminal growth and ovulate competent oocytes (7). However, sheep exhibit unique biological traits that complicate the optimization of ES protocols. For example, in prolific breeds such as the Highlander, the penultimate follicular wave contributes to double ovulations in approximately 50% of cycles (8–11). This phenomenon may be related to the inherently low estradiol production by ovine follicles (12, 59) and the shorter lifespan of the final follicular waves, which may help preserve their functional competence (9, 11, 13).

Despite growing evidence of the critical role terminal follicular development plays in fertility outcomes, most ES protocols—including fixed-time procedures—fail to address the dynamics of the ovulatory wave. Studies in cattle and sheep indicate that follicular persistence can compromise oocyte competence (14, 15). In sheep, unlike in cattle, progesterone concentrations have limited influence on follicular turnover (9, 16–18). Moreover, ovulation dispersion remains high following ES protocols—even with low-dose eCG supplementation (3, 17). This likely reflects the heterogeneity of follicular populations reaching the ovulatory stage (9).

Gonadotropin-releasing hormone (GnRH) agonists have the potential to improve the precision of ovulation control in ES protocols. Exogenous GnRH can synchronize ovulation by inducing a controlled LH surge (19). In sheep, GnRH agonists have been explored within progestogen-eCG protocols, but their effects on reproductive performance have been inconsistent (20–24, 65). These inconsistencies may result from insufficient attention to the timing of GnRH administration and the metabolic status of the animals—both factors capable of modulating the ovulatory response (21, 25).

Experimental evidence suggests that administering GnRH 30–40 h after progestogen withdrawal aligns with the natural timing of the LH surge (60), the depletion of the releasable LH pool (61), and the expected dynamics of the ovulatory wave (11, 17). In addition to improving ovulation synchrony, GnRH may reduce the risk of follicular persistence, as currently applied in cattle (Martínez-Ros and González-Bulnes, 2021). Importantly, in polyovulatory species like sheep, such interventions must also consider their impact on ovulation rate—a trait closely associated with prolificacy (7).

The Synchrovine protocol offers a valuable model for exploring how follicular wave control influences fertility. The initial PGF dose induces luteolysis and ovulation in 60–70% of treated ewes, but the resulting ovulation dispersion may hinder synchronization of subsequent follicular waves (62). Additionally, follicles from ewes that failed to undergo luteolysis can contribute to a heterogeneous population of ovulatory follicles within the group. Given the impact of follicular development on oocyte competence, ewes treated with the Synchrovine protocol are expected to exhibit reduced reproductive efficiency, as previously reported (26, 27, 62).

Thus, we hypothesized that GnRH agonist administration at strategic points during the ovulatory wave would improve ovulation synchrony while preserving follicular competence. Additionally, we postulated that GnRH could enhance the uniformity of the ovulatory follicular cohort. Accordingly, the objectives of this study were (1) to evaluate the ovulatory and fertility performance of ewes treated with GnRH after a short-term progesterone–PGF–eCG protocol, and (2) to assess the effect of GnRH-induced follicular homogenization on ovulation and fertility outcomes in ewes treated with the Synchrovine protocol.

## 2 Materials and methods

### 2.1 Animals and general management

The study involved 489 parous, non-lactating ewes (2–5 years old) and 32 sexually mature rams, from Suffolk Down, Highlander, and Suffolk × Highlander crosses. A group of 119 ewes and 10 rams (Highlander, Suffolk, and their crosses), mainly used for ovarian functional studies, were maintained at the Faculty of Veterinary Sciences, Universidad de Concepción, Chillán campus (36°S, 71°W; 124 m.a.s.l). A second group of 186 ewes and 10 rams (mainly Suffolk Down and Texel) were located at a commercial farm nearby (36°S, 71.5°W; 325 m.a.s.l.). A third group of 184 ewes and 12 rams (mainly Highlander) were kept at a commercial farm in southern Chile (40.35°S, 73°0.1 W, 79 m.a.s.l). As previously described (11), ewes at the university facility were accustomed to personnel and general management routines. They were housed in collective pens providing adequate space for resting and feeding, good ventilation, dry bedding, and ad libitum access to drinking water. During the day, ewes were allowed access to a 4-ha paddock for grazing and exercise. The diet included oat grain, commercial concentrate, and mineral salt blocks, maintaining body condition scores (BCS) around 3.0 on a 1–5 scale. The comercial flock near the university campus was managed entirely outdoors. In contrast, the southern farm housed animals in collective pens during the night, with adequate space and ventilation. At both commercial farms, ewes followed a feeding program based on ryegrass and white clover pastures (8–10 tons DM/ha/year), supplemented with oat and lupine grain (0.5 kg/ewe/day) during a 5-week flushing period before and after breeding. During winter and early spring, they were fed grass hay and mineral supplements to maintain a BCS > 2.5 at lambing and lactation. All ewes were included in a preventive health program targeting endemic diseases. Housing, management practices, and experimental procedures were approved by the Ethics Committee of the Faculty of Veterinary Sciences (CBE-20-2022), Universidad de Concepción.

### 2.2 Estrous synchronization, estrous detection, and mating programs

Estrous synchronization (ES) protocols involved either a short-term progesterone–PGF₂*α* regimen or the Synchrovine® protocol. The short-term protocol consisted of the hygienic insertion of an intravaginal progesterone-releasing device (CIDR® Sheep; 0.3 g progesterone, Cooprinsem, Osorno, Chile) for 6 days, combined with an intramuscular injection of 0.125 mg cloprostenol (Ciclase® DL, Syntex, Buenos Aires, Argentina) at CIDR removal (17). The Synchrovine® protocol involved two intramuscular doses of cloprostenol (0.125 mg each), administered 7 days apart. At the end of treatment, ewe identification numbers were marked on the flank to facilitate individual recognition during estrous detection and mating as described by Cox et al. (11) and was based on the direct observation of mating behavior in collective pens. Rams were introduced immediately after ES treatment at a ratio of 1:8–10 and rotated three times daily (08:00–09:00, 12:00–13:00, and 18:00–19:00). Ewes were considered in estrus when they stood immobile during mounting. For fertility assessments, each ewe was required to be mounted by at least two rams and a minimum of three times overall; otherwise, they were retreated with PGF₂*α* 6–7 days later. The onset of estrus was defined as the midpoint between the last rejection and the first accepted mount. The interval from PGF₂α treatment to estrus onset was defined as the treatment-to-estrus interval, while estrous response was calculated as the proportion of treated ewes that expressed behavioral estrus.

### 2.3 Follicular and corpora lutea measures and functional definitions

Ovarian ultrasonography (US) was conducted using a standardized protocol previously described (10). Antral follicles and corpora lutea (CLs) were evaluated transrectally using a 10-MHz linear-array probe attached to a real-time B-mode scanner (Honda 2010 Vet, Toyohashi, Japan). The probe was fitted to a plastic rod for transrectal manipulation, and images were viewed at ×2 magnification with constant gain and focal settings. Ovarian images were recorded, and the clearest frame was selected to measure follicular and luteal structures using internal calipers. Recruited follicles were defined as antral follicles ≥3.0 mm in diameter (28), while ovulatory-sized follicles were defined as those ≥4.3 mm, based on their ovulatory potential in Highlander ewes (10). Follicle size and position were sketched on ovary charts for later tracking. Luteal area was calculated as *π*·(diameter^2^)/4; in CLs with a cavity, the cavity area was subtracted. Ovulation was defined as the disappearance or collapse of a large follicle between two consecutive US sessions, followed by CL development in the same location 6–7 days later (functional ovulation). The time of ovulation was estimated as the midpoint between these two observations. The interval to ovulation was defined as the time (hours) between the final PGF₂*α* treatment and ovulation. Ovulation incidence was the percentage of treated ewes that ovulated, ovulation rate was the mean number of ovulations per ewe, and ovulation efficiency was the proportion of ovulatory-sized follicles that ovulated. Conception and pregnancy rates were defined as the percentage of ewes diagnosed pregnant relative to those mated and treated, respectively. Lambing rate was calculated as the percentage of ewes that lambed among those confirmed pregnant. Fecundity rate was expressed as the number of lambs born per treated ewe, while reproductive success was defined as the number of lambs born per ovulatory-sized follicle present at ovulation. Both US and behavioral assessments were performed under a double-blind protocol, with evaluators blinded to ewe treatment allocation.

### 2.4 Blood sampling and endocrine measures

Blood samples (3 mL) were collected via jugular venipuncture into heparinized glass tubes, which were immediately cooled to 5–10 °C and processed within 2 h. Plasma was separated by centrifugation at 1500 × *g* for 20 min at 5 °C, and aliquots were labeled and stored at −20 °C until analysis. Plasma progesterone concentrations were determined by solid-phase radioimmunoassay (RIA) using a commercial kit (PROG-RIA-CT, DiaSource, Louvain-la-Neuve, Belgium), previously validated for use in ruminants. The assay sensitivity was 0.05 ng/mL, with intra- and inter-assay coefficients of variation of 4.3 and 5.0%, respectively.

### 2.5 Experiments

#### 2.5.1 Experiment 1. Effect of GnRH administration on ovulation and luteal development in ewes treated with a short-term progesterone–PGF2α protocol

**Figure 1:**
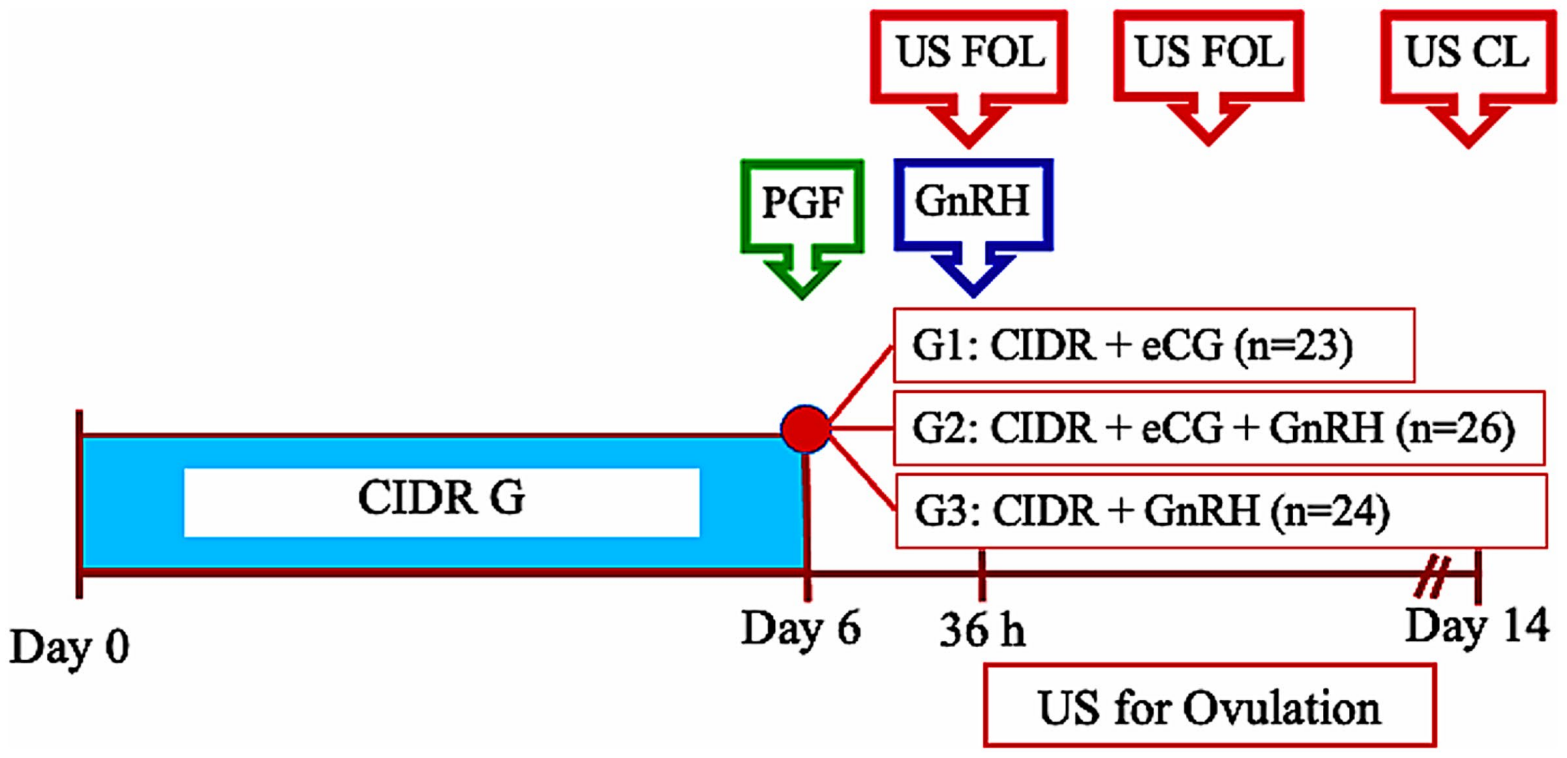
Timeline of treatments and ultrasound evaluations in Experiment 1. Ewes were synchronized with a short-term progesterone–PGF2α protocol and assigned to receive eCG and/or GnRH at indicated intervals. Ovulation was monitored every 8 h after 36 h post-CIDR removal until confirmation. US, ultrasound evaluation; FOL, follicle; CL, corpus luteum.

This experiment was conducted between May and July, within the local breeding season [February–July; (10)]. A total of 73 ewes—31 Suffolk, 18 Texel, and 24 Highlander—underwent estrous synchronization using the short-term progesterone–PGF2α protocol. At CIDR removal, animals were blocked by breed and randomly assigned to one of three treatment groups: (1) CIDR + eCG (*n* = 23), receiving 400 IU of eCG (Novormon, Syntex, Buenos Aires, Argentina) at CIDR removal; (2) CIDR + eCG + GnRH (*n* = 26), receiving 400 IU of eCG at CIDR removal and 4.2 μg of buserelin acetate (Conceptal®, MSD, Unterschleissheim, Germany) 36 h later; and (3) CIDR + GnRH (*n* = 24), receiving GnRH alone at 36 h after CIDR removal (see Figure 1). To evaluate the effect of GnRH on ovulation, ovarian ultrasonography (US) was performed at 36 h post-CIDR removal and subsequently every 8 h until ovulation was confirmed or up to 68–72 h post-treatment. A final US was conducted 7 days later to assess luteal development. Outcome variables included the number and diameter of large follicles at GnRH administration and at ovulation, the interval from treatment to ovulation, the number of corpora lutea, and total luteal area.

#### 2.5.2 Experiment 2. Effect of GnRH administration for ovulation control on post-mating reproductive performance of ewes synchronized with a short-term progesterone–PGF2α protocol

**Figure 2:**
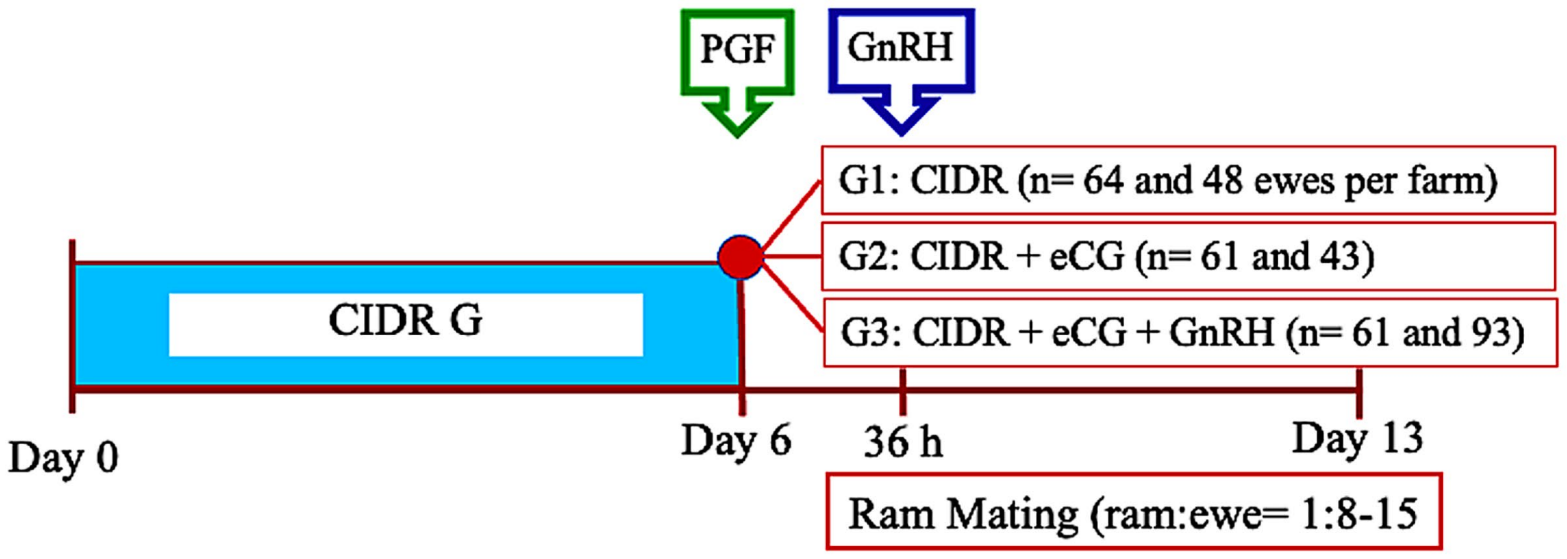
Timeline of treatments and ultrasound evaluations in Experiment 2. The scheme illustrates the distribution of ewes into experimental groups following synchronization using a short-term progesterone-PGF2α protocol.

The study was conducted between April and September (from mating to lambing) using 370 ewes and 24 mature rams from two commercial farms (*n* = 186 and 184 ewes; 10 and 14 rams, respectively; Figure 2). All ewes were synchronized using the short-term progesterone–PGF2α protocol. At CIDR removal, animals were randomly assigned to one of three groups: (1) a control group with no additional treatment (CIDR; *n* = 64 and 48 per farm), (2) a group receiving 400 IU eCG at CIDR removal (CIDR + eCG; *n* = 61 and 43), and (3) a group receiving 400 IU eCG at CIDR removal plus 4.2 μg GnRH (Conceptal®) 36 h later (CIDR + eCG + GnRH; *n* = 61 and 93). In Farm 1, rams with satisfactory breeding soundness evaluation (BSE) and marking harnesses were used for natural mating in paddocks (ram-to-ewe ratio: 1:18). Mating marks were recorded twice daily (AM and PM) as evidence of estrus, beginning immediately after CIDR removal, and rams remained for 7 days before the entire group rejoined the flock. In Farm 2, a genetic nucleus, ewes were housed in pens with controlled mating (ram-to-ewe ratio: 1:8–15), where estrous detection was conducted twice daily starting 24 h after CIDR removal. Ewes were allowed two successful mounts before being removed from the group. After 72 h, the experimental group joined the commercial flock and was exposed to clear-up rams (1:60 ratio). Pregnancy was diagnosed by transrectal ultrasonography 35–40 days post-estrus, and lambing performance was recorded in Farm 2 at 147 ± 7 days after breeding. The effects of GnRH administration on reproductive outcomes were evaluated using estrous presentation, and conception, pregnancy, lambing, and fecundity rates.

#### 2.5.3 Experiment 3. Effect of GnRH administration to control the ovulatory wave on follicular development and fertility outcomes in ewes synchronized with the Synchrovine protocol

**Figure 3:**
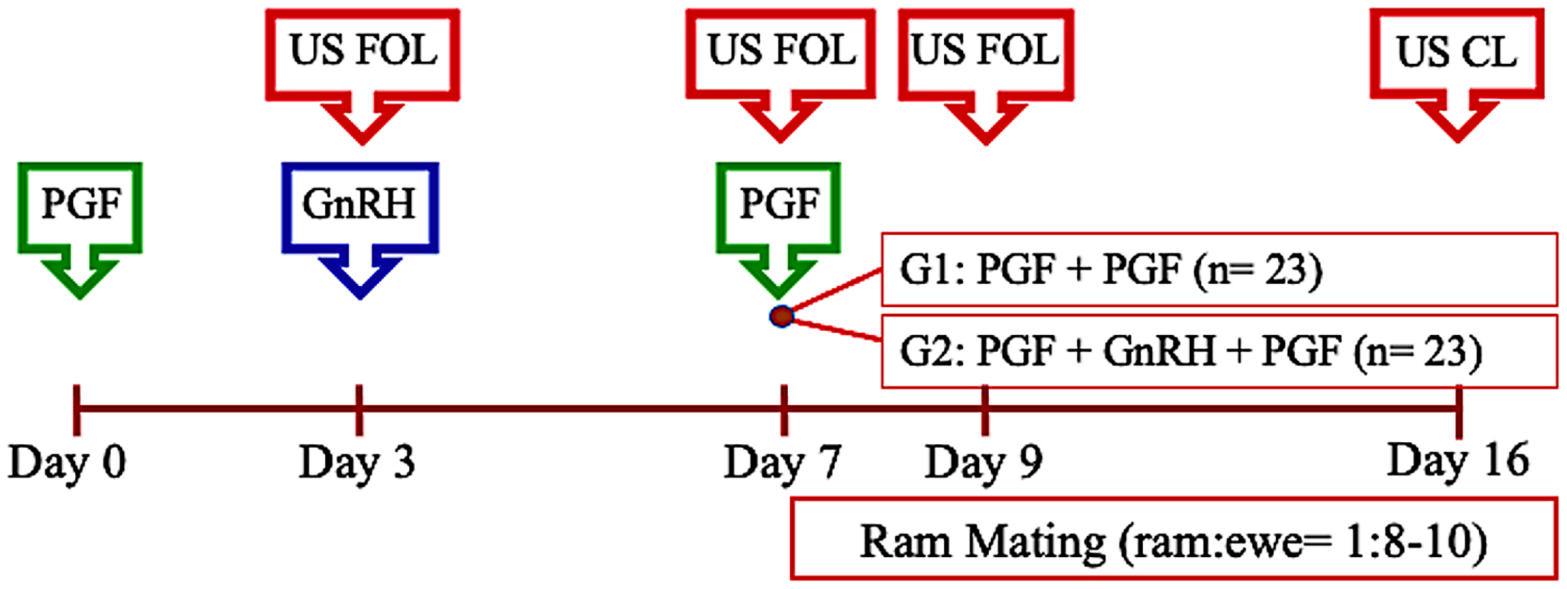
Timeline of treatments and ultrasound evaluations in Experiment 3. The scheme illustrates the distribution of ewes into experimental groups following synchronization using a synchrovine protocol.

This experiment was conducted between April and November at the university campus using 46 Highlander ewes (2–6 years old). Prior to estrous synchronization, ewes were blocked by age and randomly assigned to two groups treated with the Synchrovine® protocol. The control group received two doses of PGF₂*α* (0.125 mg DL-cloprostenol, Ciclase®) 7 days apart (PGF + PGF; *n* = 23), while the treatment group received the same protocol plus 4.2 μg buserelin acetate (Conceptal®) on Day 3 (PGF + GnRH + PGF; *n* = 23; Figure 3). The experiment was replicated three times. Ovarian follicular dynamics were evaluated by transrectal ultrasonography on Days 3 (follicles 3 days after first PGF), 7 (follicles at second PGF), and 9 (preovulatory follicles) to assess the number and diameter of antral follicles. Ovulation was evaluated on Day 11, and luteal development (CL count, luteal area, and plasma progesterone) was assessed on Day 16. Additional indicators of follicular competence included estrous expression, intervals to estrus and ovulation, and ovulation efficiency based on preovulatory-sized follicles observed on Day 9. To evaluate fertility outcomes, ewes were group-mated in collective pens using rams that rotated between pens at a ratio of 1:8–10. Pregnancy was diagnosed by ultrasonography 35 days after estrus, and lambing outcomes were recorded 147 days later. Mated ewes remained separated from rams until pregnancy diagnosis.

### 2.6 Statistical analyses

Data are expressed as means ± standard error of the mean (SEM) or as percentages, as appropriate. Normality of the data distributions was assessed using the D’Agostino–Pearson omnibus test. Parametric data were analyzed using one-way analysis of variance (ANOVA) followed by Tukey’s *post hoc* test, or Student’s *t*-test for two-group comparisons. For non-normally distributed data, the Kruskal–Wallis test followed by Dunn’s multiple comparison test or the Mann–Whitney *U* test was applied. Categorical variables, such as ovulation efficiency, pregnancy rate, and lambing rate, were evaluated using Fisher’s exact test or the Chi-square test, as appropriate. All analyses were performed using Prism software (version 10.2.3; GraphPad Software, LLC). A *p* value < 0.05 was considered statistically significant.

## 3 Results

The effect of treating ewes with GnRH to synchronize ovulations in the P_4_-PGF_2_α protocol used for ES is shown in Table 1. One ewe from the eCG-treated group was eliminated from the experiment due to a digestive disease.

**Table 1:** Effect of GnRH administration 36 h after treatment, on ovulation performance and luteal development of ewes synchronized by the 6-day CIDR-PGF_2_α protocol early in the breeding season.

Parameters	CIDR + eCG	CIDR + eCG + GnRH	CIDR + GnRH
	Mean ± SEM	Mean ± SEM	Mean ± SEM
Ewes (replicates):	24 (3)	26 (3)	24 (3)
Follicles ≥4.3 mm at 36 h^1^:			
Number (*n*):	2.3 ± 0.12^a^^2^	2.3 ± 0.17^a^	1.8 ± 0.11^b^
Diameter (mm):	5.4 ± 0.17	5.5 ± 0.14	5.8 ± 0.14
Ovulatory follicles:			
Number (*n*):	2.0 ± 0.09	1.9 ± 0.12	1.4 ± 0.14
Diameter (mm):	5.9 ± 0.14	6.0 ± 0.13	6.0 ± 0.15
Interval CIDR-ovulation (h):	63.8 ± 1.38^a^	55.5 ± 0.48^b^	56.8 ± 0.82^b^
Dev. interval to ovulation (h)^3^:	5.8 ± 0.65^a^	2.2 ± 0.36^b^	2.9 ± 0.57^b^
Ovulation Efficiency (%)	93.8 (49/54)	86.5 (50/61)	76.0 (34/45)
CL development at day 16:			
Number (*n*):	2.0 ± 0.09^a^	1.9 ± 0.12^a^	1.4 ± 0.14^b^
Total luteal area (mm):	127.3 ± 9.89	164.7 ± 14.19	141.1 ± 12.55

1 US assessment at GnRH administration.

2 Different superscripts in rows indicate significant differences (*p* < 0.05).

3 Deviation from mean ovulation interval.

GnRH administration 36 h after CIDR removal significantly reduced the treatment-to-ovulation interval (*p* < 0.0001) and narrowed the ovulation window compared to CIDR + eCG (*p* = 0.0026) and CIDR + eCG + GnRH (*p* < 0.0001). No differences were observed between the GnRH-treated groups in either interval to ovulation (*p* = 0.8482) or deviation from the mean ovulation time (*p* > 0.999).

Additionally, eCG increased the number of large follicles (≥4.3 mm) observed at 36 h post-treatment (CIDR + eCG vs. CIDR + GnRH, *p* = 0.049; CIDR + eCG + GnRH vs. CIDR + GnRH, *p* = 0.038), as well as the number of ovulated follicles (CIDR + eCG vs. CIDR + GnRH, *p* = 0.006; CIDR + eCG + GnRH vs. CIDR + GnRH, *p* = 0.014). These effects were reflected in the number of corpora lutea assessed 7 days after ovulation.

Experiment 2 evaluated the effect of GnRH administration on reproductive performance in estrous-synchronized ewes. The study was conducted on two commercial farms with distinct production objectives—lamb production (Farm 1) and genetic nucleus (Farm 2). They differed in mating precision (group mating in paddocks vs. hand mating in pens) and lambing monitoring (outdoor vs. indoor). Consequently, lambing data were only collected in Farm 2.

Results in Table 2 show that the administration of GnRH tended to increase the pregnancy rate in ewes treated with eCG only in farm 2 (*p* = 0.061). Furthermore, GnRH significantly improved fecundity rates compared to both CIDR-only (*p* = 0.007) and CIDR + eCG groups (*p* = 0.004). In contrast, no significant effects of GnRH or eCG were observed in Farm 1 (*p* > 0.10). However, when pregnancy rate following GnRH treatment was compared between farms, farm 2, based on controlled mating, exhibited significantly higher results than farm 1, based in group mating, (*p* = 0.002), suggesting that controlled mating protocols could have influenced the fertility performance. No other inter-farm differences were significant (*p* > 0.10).

**Table 2:** Effects of GnRH administration 36 h after CIDR removal on fertility and lambing performance of ewes synchronized with a 6-day CIDR-PGF2α protocol and subsequently mated.

Parameters	CIDR	CIDR + eCG	CIDR + eCG + GnRH
Farm 1:			
Ewes (*n*):	64	61	61
Estrous presentation (%):	57/64 (89.1)	57/61 (93.4)	56/61 (91.8)
Conception rate (%):	49/57 (86.0)	48/57 (84.2)	43/56 (76.8)
Pregnancy rate (%):	49/64 (76.6)	48/61 (78.7)	43/61 (70.5)^a^^1^
Farm 2:			
Ewes (*n*):	48	43	93
Estrous presentation (%):	42/48 (87.5)	41/43 (95.3)	88/93 (94.6)
Conception rate (%):	37/42 (88.1)	39/41 (95.1)	84/88 (95.5)
Pregnancy rate (%):	37/48 (77.1)	39/43 (90.7)	84/93 (90.3)^b^
Lambing rate (%):	37/37 (100)	36/39 (92.3)	79/84 (94.0)
Fecundity rate (%):	56/48 (116.7)^x^	50/43 (116.3)^x^	142/93 (152.7)^y^

1 Different superscripts in columns (a, b) and in rows (x, y) indicate statistical differences (*p* < 0.05).

Experiment 3 evaluated the effect of GnRH administration during the ovulatory wave on follicular morphology and reproductive performance in ewes synchronized with the Synchrovine protocol (Table 3).

**Table 3:** Effect of the administration of GnRH 3 days after the first dose of PGF_2_α, on morphological and functional markers of preovulatory follicle development in ewes synchronized by the synchrovine protocol during the breeding season.

Parameters	PGF + PGF Mean ± SEM	PGF + GnRH + PGF Mean ± SEM	*P*
Ewes (replicates):	23 (3)	23 (3)	
Follicles at day 3 (GnRH):			
Number (*n*):	1.9 ± 0.34	1.6 ± 0.40	0.362
Diameter (mm):	5.5 ± 0.17	5.3 ± 0.23	0.425
Deviation in diameter (mm)^1^:	0.77 ± 0.08	0.88 ± 0.14	0.788
Follicles at day 7 (PGF_2_α):			
Number (*n*):	2.3 ± 0.19	2.2 ± 0.21	0.214
Diameter (mm):	5.4 ± 0.15	5.3 ± 0.11	0.363
Deviation in diameter (mm):	0.91 ± 0.08	0.58 ± 0.06	0.004
Follicles at day 9 (preovulatory):			
Number (*n*):	2.3 ± 0.14	2.3 ± 0.13	0.565
Diameter (mm):	6.2 ± 0.14	6.4 ± 0.10	0.233
Deviation in diameter (mm):	0.73 ± 0.09	0.56 ± 0.08	0.128
Estrous presentation (%):	23/24 (95.8)	24/24 (100)	>0.999
Interval PGF-Estrus (h):	29.4 ± 1.51	27.3 ± 1.24	0.300
Interval PGF-Ovulation (h):	58.4 ± 6.54	56.5 ± 1.84	0.498
Ovulation efficiency (%)^2^:	77.8 (43/54)	92.3 (48/52)	0.061
Luteal development:			
Number (*n*):	1.8 ± 0.14	2.1 ± 0.11	0.156
Total luteal area (mm):	148.8 ± 11.53	170.9 ± 10.72	0.275
Progesterone in plasma (ng/ml):	4.6 ± 0.71	5.1 ± 0.38	0.803
Fertility markers:			
Pregnancy rate (%):	13/14 (92.9)	14/14 (100)	>0.999
Lambing rate (%):	13/13 (100)	14/14 (100)	1.0
Fecundity rate (%):	24/14 (171.4)	23/14 (164.3)	>0.999
Reproductive success (%)	66.7 (24/36)	79.3 (23/29)	0.257

1 Deviation from the mean diameter.

2 CLs/ovulatory-sized follicles at day 9.

Results showed that GnRH administration during the ovulatory wave significantly reduced follicular diameter deviation on day 7 (*p* = 0.004), indicating improved cohort homogeneity. However, no significant effects were observed on follicle number, ovulation efficiency (*p* = 0.061), luteal development, or fertility outcomes (*p* > 0.10). These results suggest that GnRH modified follicular morphology but did not improve reproductive performance.

## 4 Discussion

The main findings of this study demonstrate that administering GnRH 36 h after treatment in ewes synchronized with a short-term progesterone-PGF protocol accelerates the interval to ovulation and improves the synchrony of functionally competent ovulations. Furthermore, when GnRH is used to modulate the ovulatory follicular wave during a Synchrovine protocol, it reduces follicular diameter variability at the onset of the follicular phase, while maintaining the functional competence of oocytes for fertilization and development. These observations are consistent with the initial hypothesis. A complementary observation was that the administration of eCG increased the number of ovulatory-sized follicles and ovulation rate but an increased in fecundity rate was observed only when combined with GnRH.

The experimental model applied in this study had been validated in earlier research (10, 11), helping to minimize confounding effects, particularly those related to the metabolic influences on ovarian function (11, 29, 63) and the selection of follicular markers (10, 30, 31). Controlled mating with rams of known fertility and libido, and with appropriate ram-to-ewe ratios, further ensured reliable assessment of oocyte fertility competence.

GnRH administration 36 h after CIDR removal significantly reduced the interval to ovulation and tightened ovulatory distribution consistent with recent findings under similar conditions, including flock management and ultrasound-based monitoring (7). This outcome is conceptually expected if GnRH agonists effectively induce an LH surge (19) and there is sufficient synchronization of responsive follicles. However, studies on the use of GnRH for ovulation induction in sheep report a wide range of reproductive outcomes. Some studies support the pattern of ovulations observed here (22, 32–34) and the improved fecundity rates (23, 24), whereas others describe negative (20, 35, 36) or neutral effects on fertility (22, 32, 37). This variability likely reflects the multifactorial nature of reproductive outcomes and the diversity of experimental settings in which GnRH is tested. Research in cattle may help to identify factors that can affect the reproductive success when using GnRH in sheep.

The timing of GnRH administration is especially critical. The ovulatory wave in ruminants is regulated by LH pulsatility in coordination with metabolic hormones such as IGF-I and insulin (38). During this phase, follicles undergo terminal differentiation, including increased LH receptor expression on granulosa cells (16, 39, 40). This is an essential step for responsiveness to LH pulses and the preovulatory surge (39–41, 64). Immature follicles cannot respond reliably to premature GnRH stimulation, and the associated oocytes may lack developmental competence (25, 42). Conversely, late GnRH administration may occur after LH granules have been depleted (61), reducing the efficacy of induced surges and increasing the risk of aged follicle ovulation (8, 10).

Timing of GnRH can also be influenced by estrus detection management and ram exposure, both associated to the Male Effect (43, 44). The Male Effect, and its management, can accelerate GnRH and LH pulse frequency (45, 46), accordingly, it can also accelerate follicular development and granulosa cell differentiation, as previously discussed.

Additionally, energy balance and body condition score (BCS) play key roles in terminal follicular development and differentiation, primarily through metabolic signals, including IGF and insulin (47–49). Mechanisms controlling energy homeostasis are highly conserved and are often linked to moderate metabolic stress [(50, 51)]. However, while it is well established that energy balance impacts follicular and oocyte competence during the follicular phase, its influence on the timing of GnRH administration under subtle energy imbalances remains unclear.

In the present study, eCG increased terminal follicular growth and ovulation rate (Table 1), consistent with prior findings [reviewed by (3)]. However, no increase in fecundity rates was observed when eCG was used without GnRH (Table 2). Notably, differences in pregnancy rates between controlled and group mating systems were only observed in groups with more synchronized ovulations. While fertilization is generally not limiting in pregnancy establishment (52–54), this understanding largely derives from cattle studies. The influence of ram behavior or semen quality in sheep following synchronized ovulation remains unclear and merits further study.

The use of GnRH to synchronize the ovulatory follicular wave and reduce the incidence of ovulation from persistent follicles has been extensively studied in cattle (55, 56) but less actively in sheep (34, 57, 65). In sheep, large follicles present at the start of a follicular wave can contribute to ovulation rates in natural estrous cycles, particularly in prolific breeds (8–11, 15). However, in short-term protocols, the interval between recruitment and ovulation can be sufficiently prolonged to impair oocyte functional competence (13, 15).

Antral follicles ≥3.0 mm in diameter at the start of a follicular phase often represent a heterogeneous population (9–11). In this study, GnRH administration reduced the heterogeneity in follicular diameters within this group without affecting the ovulation efficiency or the functional competence of ovulated oocytes (Table 3). The reproductive performance of ewes treated with the Synchrovine protocol was superior to that reported after artificial insemination (26, 27, 62). This suggests that the protocol possesses an inherent potential to achieve reproductive outputs comparable to those of untreated ewes.

However, the mechanism by which GnRH affected follicle diameter remains unclear. No increase in accessory CL formation was detected at the second PGF treatment. Although persistent follicles often respond to GnRH (13, 15), the lack of luteal evidence suggests a possible suboptimal LH response, perhaps due to insufficient LH granule replenishment (61). Alternatively, GnRH might have disrupted follicular growth without promoting ovulation, consistent with findings using subovulatory doses of hCG (7, 58).

In conclusion, GnRH administration at a strategic interval from the start of the follicular phase improves the synchronization of ovulations while maintaining oocyte competence for fertilization and development. Furthermore, when used after wave emergence, GnRH effectively uniforms the diameters of the follicular cohort at the start of follicular phase, although it does not significantly impact the overall reproductive performance of treated ewes. These results highlight the importance of both timing and physiological context when using GnRH in ES protocols.

## Data Availability

The raw data supporting the conclusions of this article will be made available by the authors, without undue reservation.

## References

[1] Menchaca A Rubianes E. New treatments associated with timed artificial insemination in small ruminants. Reprod Fertil Dev. (2004) 16:403–13. doi: 10.10371/RD04037, PMID: 15315739

[2] Menchaca A Miller V Gil J Pinczac A Laca M Rubianes E. Prostaglandin F2α treatment associated with timed artificial insemination in ewe. Reprod Domest Anim. (2004) 39:352–5. doi: 10.1111/j.1439-0531.2004.00527.x 15367269

[3] González-Bulnes A Menchaca A Martin GB Martínez-Ros P. Seventy years of progestagen treatments for management of the sheep oestrous cycle: where we are and where we should go. Reprod Fertil Dev. (2020) 32:441–52. doi: 10.1071/RD18477, PMID: 31972122

[4] Yu XJ Wang J Bai YY. Estrous synchronization in ewes: the use of progestogens and prostaglandins. Acta Agriculturae Scandinavica, Sect A Anim Sci. (2019) 68:219–30. doi: 10.1080/09064702.2019.1674373

[5] Fisher JW. An economic comparison of production systems for sheep. Can J Agr Econ. (2001) 49:327–36. doi: 10.1111/j.1744-7976.2001.tb00309

[6] Snowder GD Foggarty NM. Composite trait selection to improve reproduction and ewe productivity: a review. Anim Prod Sci. (2009) 49:9–16. doi: 10.1071/EA08184

[7] Cox JF Carrasco A Navarrete F Bocic A Saravia F Dorado J. A subovulatory dose of human chorionic gonadotropin (hCG) may sustain terminal follicle development and reproductive efficiency during anestrus in sheep. Animals. (2024) 14:1096. doi: 10.3390/ani14071096, PMID: 38612335 PMC11011159

[8] Bartlewski PM Beard AP Cook SJ Chandolia RK Honaramooz A Rawlings NC. Ovarian antral follicular dynamics and their relationships with endocrine variables throughout the oestrous cycle in breeds of sheep differing in prolificacy. J Reprod Fertil. (1999) 115:111–24. doi: 10.1530/jrf.0.1150111, PMID: 10341729

[9] Bartlewski PM Sohal J Paravinja V Baby T Oliveira MEF Murawski M. Is progesterone the key regulatory factor behind ovulation rate in sheep? Domest Anim Endocrinol. (2017) 58:30–8. doi: 10.1016/j.domaniend.2016.06.006, PMID: 27639459

[10] Cox JF Jeria E Bocic A Soto-Saravia R Dorado J Saravia F. Characterization of the productive performance of highlander sheep in southern Chile. I. Female reproductive traits. Small Rumin Res. (2015) 130:183–8. doi: 10.1016/j.smallrumres.2015.06.010

[11] Cox JF Navarrete F Carrasco A Dorado J Saravia F. Effect of bST administration on plasma concentrations of IGF-I and follicular dynamics and ovulation during the interovulatory cycle of sheep and goats. Theriogenology. (2019) 123:159–66. doi: 10.1016/j.theriogenology.2018.10.003, PMID: 30308392

[12] Goodman RL Inskeep EK. Neuroendocrine control of the ovarian cycle in the sheep In: Neill JD, editor. Knobil and Neill’s physiology of reproduction. 3rd ed. New York: Academic Press (2006). 2389–447.

[13] Mihm M Baguisi A Boland MP Roche JF. Association between the duration of dominance of the ovulatory follicle and pregnancy rate in beef heifers. J Reprod Fertil. (1994) 102:123–30. doi: 10.1530/jrf.0.1020123, PMID: 7799304

[14] Mihm M Curran N Hyttel P Knight PG Boland MP Roche JF. Effect of dominant follicle persistence on follicular fluid oestradiol and inhibin and on oocyte maturation in heifers. J Reprod Fertil. (1999) 116:293–304. doi: 10.1530/jrf.0.1160293, PMID: 10615254

[15] Seekallu S Toosi B Grazul-Bilska AT Rawlings NC. Markers of ovarian antral follicular development in sheep: comparison of follicles destined to ovulate from the final or penultimate follicular wave of the estrous cycle. Reproduction. (2010) 140:559–68. doi: 10.1530/REP-10-0064 20634390

[16] Abreu FM Coutinho da Silva MA Cruppe LH Mussard ML Bridges GA Harstine BR. Role of progesterone concentrations during early follicular development in beef cattle: I. Characteristics of LH secretion and oocyte quality. Anim Reprod Sci. (2018) 196:59–68. doi: 10.1016/j.anireprosci.2018.06.020, PMID: 30149874

[17] Cox JF Allende R Lara E Leiva A Díaz T Dorado J. Follicular dynamics, interval to ovulation and fertility after AI in short-term progesterone and PGF2α oestrous synchronization protocol in sheep. Reprod Domest Anim. (2012) 47:946–51. doi: 10.1111/j.1439-0531-2012.01996.x 22471421

[18] Savio JD Thatcher WW Morris GR Entwistle K Drost M Mattiacci MR. Effect of induction of low plasma progesterone concentrations with a progesterone-releasing device on follicular turnover and fertility in cattle. J Reprod Fertil. (1993) 98:77–84. doi: 10.1530/jrf.0.0980077 8345482

[19] Goodman RL Herbison AE Lehman MN Navarro VM. Neuroendocrine control of gonadotropin-releasing hormone: pulsatile and surge modes of secretion. J Neuroendocrinol. (2022) 34:e13094. doi: 10.1111/jne.13094, PMID: 35107859 PMC9948945

[20] dos Santos A Zandonadi F García LA Ferreira J. Effects of GnRH administration on ovulation and fertility in ewes subjected to estrous synchronization. R Bras Zootec. (2012) 41:1412–8. doi: 10.1590/S1516-35982012000600014

[21] Olivera-Muzante J Gil J Viñoles C Fierro S. Reproductive outcome with GnRH inclusion at 24 or 36h following a prostaglandin F2α-based protocol for timed AI in ewes. Anim Reprod Sci. (2013) 138:175–9. doi: 10.1016/j.anireprosci.2013.02.013, PMID: 23537480

[22] Reyna J Thomson PC Evans G Maxwell WMC. Synchrony of ovulation and follicular dynamics in merino ewes treated with GnRH in the breeding and non-breeding seasons. Reprod Domest Anim. (2007) 42:410–7. doi: 10.1111/.1439-0531.2006.00800.x 17635779

[23] Sirjani MA Kohram H Shahir MH. Effects of eCG injection combined with FSH and GnRH treatment on the lambing rate in synchronized Afshari ewes. Small Rumin Res. (2012) 106:59–63. doi: 10.1016/j.smallrumres.2012.04.022

[24] Türk G Gür S Sonmez M Bozkurt T Aksu EH Aksoy H. Effect of exogenous GnRH at the time of artificial insemination on reproductive performance of Awassi ewes synchronized with progestagen-PMSG-PGF2alpha combination. Reprod Domest Anim. (2008) 43:308–13. doi: 10.1111/j.1439-0531.2007.00896.x, PMID: 18067532

[25] Atkins JA Smith MF Wells KJ Geary TW. Factors affecting preovulatory follicle diameter and ovulation rate after gonadotropin-releasing hormone in postpartum beef cows. Part I: cycling cows. J Anim Sci. (2010) 88:2300–10. doi: 10.2527/jas.2009-2532 20228240

[26] Fierro S Gil J Viñoles C Olivera-Muzante J. The use of prostaglandins in controlling estrous cycle of the ewe: a review. Theriogenology. (2013) 79:399–408. doi: 10.1016/j.theriogenology.2012.10.022, PMID: 23219520

[27] Vilariño M Rubianes E Menchaca A. Ovarian responses and pregnancy rate with previously used intravaginal progesterone releasing devices for fixed-time artificial insemination in sheep. Theriogenology. (2013) 79:206–10. doi: 10.1016/j.theriogenology.2012.10.007, PMID: 23127920

[28] McNatty KP Heath DA Hudson NL Reader KL Quirke L Lun S. The conflict between hierarchical ovarian follicular development and superovulation treatment. Reproduction. (2010) 140:287–94. doi: 10.1530/REP-10-0165, PMID: 20501789

[29] Scaramuzzi TJ Campbell BK Downing JA Kendall NR Khalid M Muñoz-Gutiérrez M. A review of the effects of supplementary nutrition in the ewe on the concentrations of reproductive and metabolic hormones and mechanisms that regulate folliculogenesis and ovulation rate. Reprod Nutr Dev. (2006) 46:339–54. doi: 10.1051/rnd:2006016 16824444

[30] Ginther OJ. How ultrasound technologies have expanded and revolutionized research in reproduction in large animals. Theriogenology. (2014) 81:112–25. doi: 10.1016/j.theriogenology.2013.09.007, PMID: 24274416

[31] Seekallu SV Toosi BM Duggavathi R Barrett DMW Davies KL Waldner C. Ovarian antral follicular dynamics in sheep revisited: comparison among estrous cycles with three or four follicular waves. Theriogenology. (2010) 73:670–80. doi: 10.1016/j.theriogenology.2009.11.007, PMID: 20034659

[32] Martínez-Ros P González-Bulnes A. Efficiency of CIDR-based protocols including GnRH instead of eCG for estrous synchronization in sheep. Animals. (2019) 9:146. doi: 10.3390/ani9040146, PMID: 30987248 PMC6523624

[33] Silva BDM Silva TASN Moreira NH Teixeira HCA Paiva Neto MA Neves JP. Ovulation induction in ewes using GnRH in long and short-term synchronization protocol. Anim Reprod. (2015) 12:312–5.

[34] Yu X Bai Y Yang J Zhao X Zhang L Wang J. Comparison of five protocols of estrous synchronization on reproductive performance of Hu sheep. Front Vet Sci. (2022) 9:843514. doi: 10.3389/fvets.2022.843514, PMID: 35464353 PMC9019657

[35] Sun S Yang N Zhang J Wu X Liu Y Li X. Effects of exogenous GnRH administration at insemination on pregnancy rates of estrus-synchronized seven ewe populations during the breeding season. Anim Reprod. (2025) 22:e20240085. doi: 10.1590/1984-3143-AR2024-0085, PMID: 40013123 PMC11864727

[36] Zhang J Sun S Bai X Yang N Liu Y Wu X. Metabolomics analysis of the effect of GnRH on the pregnancy rate of ewes with estrus synchronization scheme based on progesterone. Front Vet Sci. (2024) 11:1442931. doi: 10.3389/fvets.2024.1442931, PMID: 39055862 PMC11270128

[37] Zonturlu AK Kaçar C Kaya S Emre B Korkmaz Ö Ari UÇ. Effect of double GnRH injections on reproductive parameters in Awassi ewes receiving long-term progesterone. J Appl Anim Res. (2018) 46:1103–7. doi: 10.1080/09712119.2018.1469497

[38] Scaramuzzi TJ Baird DT Campbell BK Driancourt M-A Dupont J Fortune JE. Regulation of folliculogenesis and the determination of ovulation rate in ruminants. Reprod Fertil Dev. (2011) 23:444–67. doi: 10.1071/RD09161 21426863

[39] Campbell BK Kendall NR Baird DT. The effect of the presence and pattern of luteinizing hormone stimulation on ovulatory follicle development in sheep. Biol Reprod. (2007) 76:719–27. doi: 10.1095/biolreprod.106.053462, PMID: 17167168

[40] Luo W Gunmen A Haughian JM Wiltbank MC. The role of luteinizing hormone in regulating gene expression during selection of a dominant follicle in cattle. Biol Reprod. (2011) 84:369–78. doi: 10.1095/biolreprod.110.085274 20962252

[41] Moorey SE Hessock EA Edwards JL. Preovulatory follicle contributions to oocyte competence in cattle: importance of the ever-evolving intrafollicular environment leading up to the luteinizing hormone surge. J Anim Sci. (2022) 100:skac153. doi: 10.1093/jas/skac153, PMID: 35772757 PMC9246662

[42] Geary TW Smith MF MacNeil MD Day ML Bridges GA Perry GA. Influence of follicular characteristics at ovulation on early embryonic survival. J Anim Sci. (2013) 91:3014–21. doi: 10.2527/jas.2012-5887 23230106

[43] Delgadillo JA Hernández H Abecia JA Keller M Chemineau P. Is it time to reconsider the relative weight of sociosexual relationships compared with photoperiod in the control of reproduction of small ruminant females? Domest Anim Endocrinol. (2020) 73:106468. doi: 10.1016/j.domaniend.2020.106468, PMID: 32249000

[44] Fabre-Nys C Chanvallon A Dupont J Lardic L Lomet D Martinet S. The "ram effect": a "non-classical" mechanism for inducing LH surges in sheep. PLoS One. (2016) 11:e0158530. doi: 10.1371/journal.pone.0158530, PMID: 27384667 PMC4934854

[45] Delgadillo JA Gelez H Ungerfeld R Hawken PAR Martin GB. The ‘male effect’ in sheep and goats—revisiting the dogmas. Behav Brain Res. (2009) 200:304–14. doi: 10.1016/j.bbr.2009.02.004, PMID: 19374015

[46] Hawken PAR Beard AP Esmaili T Kadokawa H Evans ACO Blache D. The introduction of rams induces an increase in pulsatile LH secretion in cyclic ewes during the breeding season. Theriogenology. (2007) 68:56–66. doi: 10.1016/j.theriogenology.2007.03.023, PMID: 17477966

[47] Ipsa E Cruzat VF Kagize JN Yocich JL Keane KN. Growth hormone and insulin-like growth factor action in reproductive tissues. Tissues Front Endocrinol. (2019) 10:777. 2019. doi: 10.3389/fendo.2019.00777 PMC6861326 31781044

[48] Meikle A de Brun V Carriquiry M Soca P Sosa C Adrien ML. Influences of nutrition and metabolism on reproduction of the female ruminant. Anim Reprod. (2018) 15:899–911. doi: 10.21451/1984-3143-AR2018-0017 36249854 PMC9536053

[49] Price CA Estienne A. The life and death of the dominant follicle. Anim Reprod. (2018) 15:680–90. doi: 10.21451/1984-3143-AR2018-0030, PMID: 36249855 PMC9536057

[50] Gross JJ Bruckmaier RM. Invited review: metabolic challenges and adaptation during different functional stages of the mammary gland in dairy cows: perspectives for sustainable milk production. J Dairy Sci. (2019) 102:2828–43. doi: 10.3168/jds.2018-15713, PMID: 30799117

[51] Walsh SW Matthews D Browne JA Forde N Crowe MA Diskom M. Acute dietary restriction in heifers alters expression of genes regulating exposure and response to gonadotrophins and IGF in dominant follicles. Anim Reprod Sci. (2012) 133:43–51. doi: 10.1016/j.anireprosci.2012.06.012 22771244

[52] Dalton JC. Insemination related factors affecting fertilization in estrous-synchronized cattle In: Proceeding applied reproductive strategies in beef cattle, October 8–9, 2014, Stillwater, Oklahoma (2014). 169–85.

[53] Smith MF Pohler KG Perry GA Patterson D. Physiological factors that affect pregnancy rates to artificial insemination in beef cattle In: Proceeding applied reproductive strategies in beef cattle, December 3-4, Sioux Falls, SD (2012). 33–51.

[54] Utt MD Day ML. The obstacle course to successful establishment of pregnancy in domestic livestock species. J Anim Sci. (2013) 91:2993–9. doi: 10.2527/jas.2012-5947 23345560

[55] Hassanein EM Szelényi Z Szenci O. Gonadotropin-releasing hormone (GnRH) and its agonists in bovine reproduction I: structure, biosynthesis, physiological effects, and its role in estrous synchronization. Animals. (2024) 14:1473. doi: 10.3390/ani14101473, PMID: 38791690 PMC11117390

[56] Sartori R Consentini CEC Alves RLOR Silva LO Wiltbank MC. Review: manipulation of follicle development to improve fertility of cattle in timed-artificial insemination programs. Animal. (2023) 17:100769. doi: 10.1016/j.animal.2023.100769, PMID: 37567674

[57] Karaca F Ataman MB Coyan K. Synchronization of estrus with short- and long-term progestagen treatments and the use of GnRH prior to short-term progestagen treatment in ewes. Small Rumin Res. (2009) 81:185–8. doi: 10.1016/j.smallrumres.2008.12.002

[58] Bruno-Galarraga M Cano-Moreno V Lago-Cruz B Encinas T González-Bulnes A Martínez-Ros P. The use of hCG for inducing ovulation in sheep estrus synchronization impairs ovulatory follicle growth and fertility. Animals. (2021) 11:984. doi: 10.3390/ani11040984, PMID: 33915793 PMC8065977

[59] Dobson H Campbell BK Gordon BM Scaramuzzi RJ. Endocrine activity of induced persistent follicles in sheep. Biol Reprod. (1997) 56:208–213. doi: 10.1095/biolreprod56.1.208 9002651

[60] Van Cleeff J Karsh FJ Padmanabhan V. Characterization of endocrine events during the periestrous period in sheep after estrous synchronization with controlled internal drug release (CIDR) device. Domest Anim Endocrinol. (1998) 15:23–34. doi: 10.1016/s0739-7240(97)00059-3 9437582

[61] McNeilly AS Crawford JL Taragnat C Nicol L McNeilly JR. The differential secretion of FSH and LH: regulation through genes, feedback and packaging. Reproduction (Supplement 61). (2003) 463–471. 14635955

[62] Vilariño M Cuadro F dos Santos-Neto PC García-Pintos C Menchaca A. Time of ovulation and pregnancy outcomes obtained with Synchrovine for FTAI in sheep. Theriogenology. (2017) 90:163–168. doi: 10.1016/j.theriogenology.2016.12.003 28166963

[63] Robinson JJ Ashworth CJ Rooke JA Mitchell LM McEvoy TG. Nutrition and fertility in ruminant livestock. Anim Feed Sci Tech. (2006) 126:259–276. doi: 10.1016/j.anifeedsci.2005.08.006

[64] Gong JG Campbell BK Webb R. Defining the gonadotrophin requirement for the selection of a single dominant follicle in cattle. Reprod Fertil Dev. (2020) 32:322–334. doi: 10.1071/RD19060 31656220

[65] Martinez-Ros P Astiz S Garcia-Rosello E Rios-Abellan A Gonzalez-Bulnes A. Effects of short-term intravaginal progestagens on the onset and features of estrus, preovulatory LH surge and ovulation in sheep. Anim Reprod Sci. (2018) 197:317–323. doi: 10.1016/j.anireprosci.2018.08.046 30201256

